# Adrenergic regulation during acute hepatic infection with *Entamoeba histolytica* in the hamster: involvement of oxidative stress, Nrf2 and NF-KappaB

**DOI:** 10.1051/parasite/2017048

**Published:** 2017-11-29

**Authors:** Liseth Rubi Aldaba-Muruato, Martín Humberto Muñoz-Ortega, José Roberto Macías-Pérez, Julieta Pulido-Ortega, Sandra Luz Martínez-Hernández, Javier Ventura-Juárez

**Affiliations:** 1 Departamento de Morfología, Universidad Autónoma de Aguascalientes, Aguascalientes, Ags. México; 2 Departamento de Química, Centro de Ciencias Básicas, Universidad Autónoma de Aguascalientes, Aguascalientes, Ags. México; 3 Química Clínica, Unidad Académica Multidisciplinaria Zona Huasteca, Universidad Autónoma de San Luis Potosí, Ciudad Valles, SLP. México

**Keywords:** Adrenergic regulation, oxidative stress, Nrf2, NF-kappa-B, *Entamoeba histolytica*, amebic liver abscess

## Abstract

Oxidative stress and transcriptional pathways of nuclear factor erythroid 2-related factor 2 (Nrf2) and nuclear factor kappa-B (NF-κB) are critically involved in the etiopathology of amebic liver abscess (ALA). In this work, we studied the relationship between the adrenergic nervous system and ALA in the hamster. ALA was visible at 12 h of infection. While 6-hydroxidopamine (6-OHDA) decreased infection, propranolol (β-adrenergic blocker) treatment was associated with less extensive liver damage, and phentolamine treatment (α-adrenergic blocker) significantly reduced ALA compared to 6-OHDA and propranolol. Serum enzymatic activities of alanine aminotransferase (ALT) and γ-glutamyl transpeptidase (γ-GTP) were increased at 12 h post-infection. Chemical denervation and α and β-adrenergic blockers decreased ALT to normal levels, while 6-OHDA and propranolol showed a trend to decrease γ-GTP but phentolamine significantly reduced γ-GTP. Amebic infection increased oxidized glutathione (GSSG) and decreased both reduced glutathione (GSH) and the GSH/GSSG ratio. Propranolol and 6-OHDA showed a tendency to decrease GSSG. However, GSH, GSSG and GSH/GSSG returned to normal levels with phentolamine. Furthermore, amebic infection increased pNF-κB and interleukin-1β (IL-1β), and showed a tendency to decrease hemoxigenase-1 (HO-1), but not Nrf2. Chemical denervation showed a trend to decrease pNF-κB and IL-1β, and neither Nrf2 nor HO-1 increased significantly. In addition, NF-κB and IL-1β were attenuated by propranolol and phentolamine treatments, although phentolamine showed significant overexpression of Nrf2 and HO-1. This suggests that the adrenergic system may be involved in oxidative stress and in modulation of the Nrf2 and NF-κB pathways during ALA development.

## Abstract (fr)

Le stress oxydatif et les voies transcriptionnelles du facteur 2 lié au facteur nucléaire érythroïde 2 (Nrf2) et au facteur nucléaire kappa-B (NF-κB) sont fortement impliqués dans l'étiopathologie de l'abcès hépatique amibien (AHA). Dans ce travail, nous avons étudié les relations entre le système nerveux adrénergique et l'AHA chez le hamster. L'AHA était visible à 12 h d'infection. Alors que la 6-hydroxydopamine (6-OHDA) a diminué l'infection, le traitement au propranolol (bloquant β-adrénergique) était associé à des lésions hépatiques moins importantes et le traitement par phentolamine (α-bloquant) a réduit significativement l'AHA par rapport au 6-OHDA et au propranolol. Les activités enzymatiques sériques de l'alanine aminotransférase (ALT) et de la γ-glutamyl transpeptidase (γ-GTP) ont été augmentées 12 h après l'infection. La dénervation chimique et les bloqueurs α et β-adrénergiques ont diminué les taux d'ALT à des niveaux normaux, tandis que la 6-OHDA et le propranolol ont montré une tendance à la diminution du γ-GTP mais que la phentolamine a significativement réduit le γ-GTP. L'infection amibienne a augmenté le glutathion oxydé (GSSG) et diminué à la fois le glutathion réduit (GSH) et le rapport GSH/GSSG. Le propranolol et la 6-OHDA ont montré une tendance à la diminution de GSSG. Cependant, GSH, GSSG et GSH/GSSG sont revenus à des niveaux normaux avec la phentolamine. En outre, l'infection amibienne a augmenté pNF-κB et interleukine-1β (IL-1β) et a montré une tendance à diminuer hémoxigénase-1 (HO-1), mais pas Nrf2. La dénervation chimique a montré une tendance à diminuer pNF-κB et IL-1β, et ni Nrf2 ni HO-1 n'ont augmenté de manière significative. De plus, le NF-κB et l'IL-1β ont été atténués par les traitements au propranolol et à la phentolamine, bien que la phentolamine ait montré une surexpression significative de Nrf2 et de HO-1. Ceci suggère que le système adrénergique peut être impliqué dans le stress oxydatif et dans la modulation des voies Nrf2 et NF-κB durant le développement de l'AHA.

## 1 Introduction

*Entamoeba histolytica* causes amebic liver abscess (ALA) as a result of the perforation of the large intestine and liver invasion [23,46]; this parasite affects about 500 million people worldwide [2]. The focal destruction of the liver is attributed mainly to parasite pathogenicity factors and the massive early accumulation of neutrophils, inflammatory monocytes and macrophages [24]. This inflammatory response after amebic infection is considered crucial to the pathogenesis and establishment of the lesion, in spite of the inherent virulence of the parasite [9]. In addition, many studies have suggested that there is a connection between inflammatory diseases and the central nervous system (CNS) [15,58]. The CNS has been reported to regulate the immune system through two pathways: first, hormonal response, including the hypothalamic-pituitary-adrenal axis, as well as the hypothalamic-pituitary-gonadal, the hypothalamic-pituitary-thyroid and the hypothalamic-growth-hormone axes; and second, the autonomic nervous system through the release of norepinephrine and acetylcholine from sympathetic and parasympathetic nerves [15]. Due to these observations, researchers have begun to study the involvement of the autonomic nervous system during the inflammatory response induced by *E. histolytica,* suggesting that the sympathetic and parasympathetic nervous systems (SNS, PNS, respectively) modify immune response to amebic infection, inducing deregulation of pro-inflammatory proteins, such as NF-κB [3,41,51]. Moreover, recently we demonstrated that during the acute phase of ALA, the pro-inflammatory system of NF-κB is activated while the host antioxidant system of Nrf2 is decreased, inducing uncontrolled inflammation and oxidative stress. This contributes to increased disease severity [1]. Nrf2 is a transcriptional factor that controls the antioxidant defense system through induction of several stress response proteins, such as HO-1 [37]. Therefore, the present study was designed to investigate the possible regulation role of the SNS on oxidative stress, NF-κB and Nrf2 in acute amebic infection of the hamster liver.

## 2 Materials and Methods

### 2.1 Ethics

All animals were treated humanely at the animal facilities of the Autonomous University of Aguascalientes and in accordance with the guidelines of the Committee on Bioethics, which are based on the NIH guidelines for animal research [43].

### 2.2 Animals

Male golden hamsters (*Mesocricetus auratus*) of 140-160 g body weight were used in this study. They were maintained on a standard diet of Purina Chow with free access to drinking water.

### 2.3 List of chemicals

6-OHDA hydrobromide, DL-propranolol hydrochloride, phentolamine hydrochloride, guanidine hydrochloride, DL alanine, α-ketoglutaric acid, sodium pyruvate, 2,4-dinitrophenilhydrazine, metaphosphoric acid, 2-propanol and 1-bromo-3-chloropropane (Sigma Chemical, St. Louis, MO, USA).

### 2.4 Intrahepatic amebic infection

The trophozoites of *E. histolytica* strain HM-1:IMSS were passed multiple times through animal livers to maintain virulence and were grown under axenic conditions in Diamond's TYI-S-33 medium at 36°C, according to the Diamond procedure [14]. The hamster liver was inoculated with the virulent amebic trophozoites in the exponential phase of growth (5 × 10^5^) with a volume of 100 μL culture medium, as previously described [62,63]. The surgical procedure for hepatic amebiasis infection was carried out in anesthetized animals with sodium pentobarbital (30 mg/kg, i.p.).

### 2.5 Experimental design

We designed four amebic experimental groups, and seven healthy control groups:

*Amebic experimental groups:* one group of hamsters was inoculated only with *E. histolytica* trophozoites (ALA group, n=5). A second was treated with 6-OHDA (40 mg/kg, i.p.) daily for 10 days before amebic inoculation (6-OHDA + ALA group, n=5). A third and fourth group were administered either propranolol (10 mg/kg, i.p.) daily for 2 days prior to the day of inoculation (propranolol + ALA group, n=5) or phentolamine (10 mg/kg, i.p.) daily for 2 days prior to the day of inoculation and 40 minutes before amebic infection (phentolamine + ALA group, n=5). All animals were sacrificed 12 h after amebic infection. Drugs doses were reported previously: 6-OHDA [3] and propranolol and phentolamine [39].

*Healthy control groups:* the intact group included animals neither treated nor with sham surgery (intact group, n=5). A sham-operated group received 100 μL of Diamond medium without trophozoites (sham group, n=5). The 6-OHDA comparator group was treated with 6-OHDA (40 mg/kg, i.p. daily for 10 days, n=5) before sham operation. The propranolol comparator group received propranolol (10 mg/kg, i.p. daily for 2 days, n=5) before sham operation. The phentolamine comparator group was treated with phentolamine (10 mg/kg, i.p. daily, n=5) 2 days before sham operation and again 40 minutes before sham surgery. An additional hamster group was administered 0.9% saline solution plus 0.01 % ascorbic acid (vehicle 6-OHDA, i.p., daily for 10 days) and sham-operated (vehicle 1 group, n=5) and the other group was given 0.9% saline solution (vehicle of α- and β-adrenergic blocking agents) daily for 2 days, and 40 minutes before sham surgery (vehicle 2 group, n=5).

### 2.6 Sacrificed animals

Animals were anesthetized with sodium pentobarbital (50 mg/kg, i.p.). Blood was collected *via* cardiac puncture and livers were carefully dissected, freed from surrounding fatty and fibrous tissues, and immediately rinsed with 0.9% saline solution. Tissue was snap frozen in liquid nitrogen and then stored at −20°C until use. Fragments of liver with damaged areas were taken and fixed in 4% paraformaldehyde in phosphate buffered saline (PBS).

### 2.7 Evaluation of the autonomic nervous system: heart rate (HR), hepatic tyrosine hydroxylase (TH) synthesis and liver glycogen content

HR measurement is a non-invasive, practical and reproducible measure of autonomic nervous system function. Pulse oximeters process biological signals in a safe and precise way and monitor HR and oxygen saturation [4,30] in real-time. In this work, we used a pulse oximeter (CMS60D, Lake Bluff, IL, USA) that was attached to the left crotch to measure HR before treatments, surgery and sacrifice procedures. In addition, 6-OHDA is widely used to lesion the nigrostriatal dopaminergic system as a model for Parkinson's disease [61]. It is well known that 6-OHDA destroys catecholaminergic neurons through the combined action of reactive oxygen species (ROS) and quinones [8]. Animals were experimentally sympathectomized with 6-OHDA (40 mg/kg, for 7 days); this substance acts as a false neurotransmitter, destroying the terminal sympatethic nerves by forming oxygen reactive species, thus inhibiting TH function for adrenaline and noradrenaline synthesis, in axonal fibers surrounding the blood vessels in the liver [12,42]. In the present work, immunohistochemical staining of TH was used to study the depletion of dopaminergic neurons in the liver tissue [50]. Briefly, the primary antibody was a rabbit polyclonal anti-TH (5 μg/mL; Sigma Aldrich SAB4502966). The immunohistochemical staining was developed using the avidin-biotin, peroxidase complex (1:10: ABC Kit; Vector Laboratories; Burlingame, CA, USA) and 3,3-diaminobenzidine (DAB; Sigma-Aldrich; St. Louis, MO, USA). Tissue samples were mounted and pictures were taken using a microscope with the same camera configuration and light intensity for each slice. Moreover, liver glycogen content was evaluated because glycogen is the main source of energy in the body and the hepatic content of this carbohydrate is an indicator of metabolism and functionality [53]. Glycogen metabolism is also regulated by a classical β-adrenergic receptor-adenylyl cyclase system [16]. Therefore, small liver samples with amebic abscess (0.1 g) were separated for glycogen determination using the anthrone reagent [53].

### 2.8 Hematoxylin and eosin (H&E) staining

To visualize ALA development, we performed H&E staining as described in the Manual of Histologic Staining Methods of the Armed Forces [36]. In addition, each ALA sample from infected hamsters was investigated to determine the percentages of necrotic and inflammatory areas compared with healthy controls, using ImageJ software.

### 2.9 Serum activity of alanine aminotransferase (ALT) and γ-glutamyl transpeptidase (γ-GTP)

ALT is an enzyme stored in the cytosol of hepatocytes, and when these are damaged or destroyed, the enzyme escapes to the systemic circulation, and levels in the serum are widely recognized as a very important indicator to judge the severity of acute hepatic injury [7]. Furthermore, γ-GTP is an enzyme embedded in the hepatocyte plasma membrane, mainly in the canalicular domain. Therefore, serum was used to determine liver damage by measuring ALT [49] and γ-GTP [20].

### 2.10 Reduced glutathione (GSH) and oxidized glutathione (GSSG)

Oxidative stress is commonly associated with several liver diseases including ALA [1,55]. Therefore, we measured intracellular reduced glutathione (GSH) and oxidized glutathione (GSSG) in liver samples using an EnzyChrom^TM^ GSH/GSSG Assay Kit, (Bioassay Systems, Hayward, CA, USA).

### 2.11 Isolation of total proteins

TRI Reagent^®^ (Sigma-Aldrich T9424) was used to isolate total protein of liver samples (50 mg) from healthy animals and samples of ALA lesion from infected animals, which were obtained by macroscopic observations. Total protein was determined by the Bradford method [5].

### 2.12 Western blot assays

Volumes equivalent to 50 µg of total proteins were transferred to a 12% polyacrylamide gel; separated proteins were transferred to an Immuno-Blot^TM^ PVDF membrane (BioRad, Hercules, CA, USA). Next, blots were blocked with 5% skim milk and 0.05% Tween-20 for 1 h at room temperature and independently incubated at room temperature with antibodies that are selective for each protein, phosphor NF-κB Ser536 (Cell Signaling 3033), heme oxygenase-1 (HO-1) and Nrf2 (LifeSpan BioSciences LS-C15743, LS-C154863, respectively) and Interleukin-1β (IL-1β) (Millipore, MAB1001). Membranes were washed and then exposed for 1 h at room temperature to anti-mouse, anti-goat and anti-rabbit IgG (Sigma A9044, A5420, A0545) respectively, diluted 1:2,000 in the blocking solution. Blots were washed and protein developed using the Clarity Western ECL Substrate (Bio-rad, 170-5061). Blots were incubated with a monoclonal antibody directed against β-actin (Sigma, A2066), which was used as a control to normalize protein production levels. The procedure to strip membranes was as follows. First, blots were washed four times with phosphate-saline buffer pH 7.4 (0.015 M, 0.9% NaCl), then immersed in stripping buffer (2-mercaptoethanol 100 mM, sodium dodecyl sulfate 2% and Tris–HCl 62.5 mM, pH 6.7) for 30 min at 60°C with gentle shaking. Membranes were then washed five times with 0.05% Tween-20 in phosphate saline buffer. The protein expressions were analyzed densitometrically using ImageJ software.

### 2.13 Statistical analyses

Data are expressed as mean values ± SE. Comparisons were carried out by analysis of variance followed by Tukey's test, as appropriate, using GraphPad Prism 5.00 software. Differences were considered statistically significant when *p*<0.05.

### 2.14 Photographic images

Images were taken with a Cool Snap Pro camera associated with a phase contrast Carl Zeiss Axioscop 40 microscope, and the images were processed with Media Cybernetic Image Pro Plus software.

## 3 Results

### 3.1 Evaluation of the autonomic nervous system: HR, hepatic TH synthesis and glycogen content

**Figure 1:**
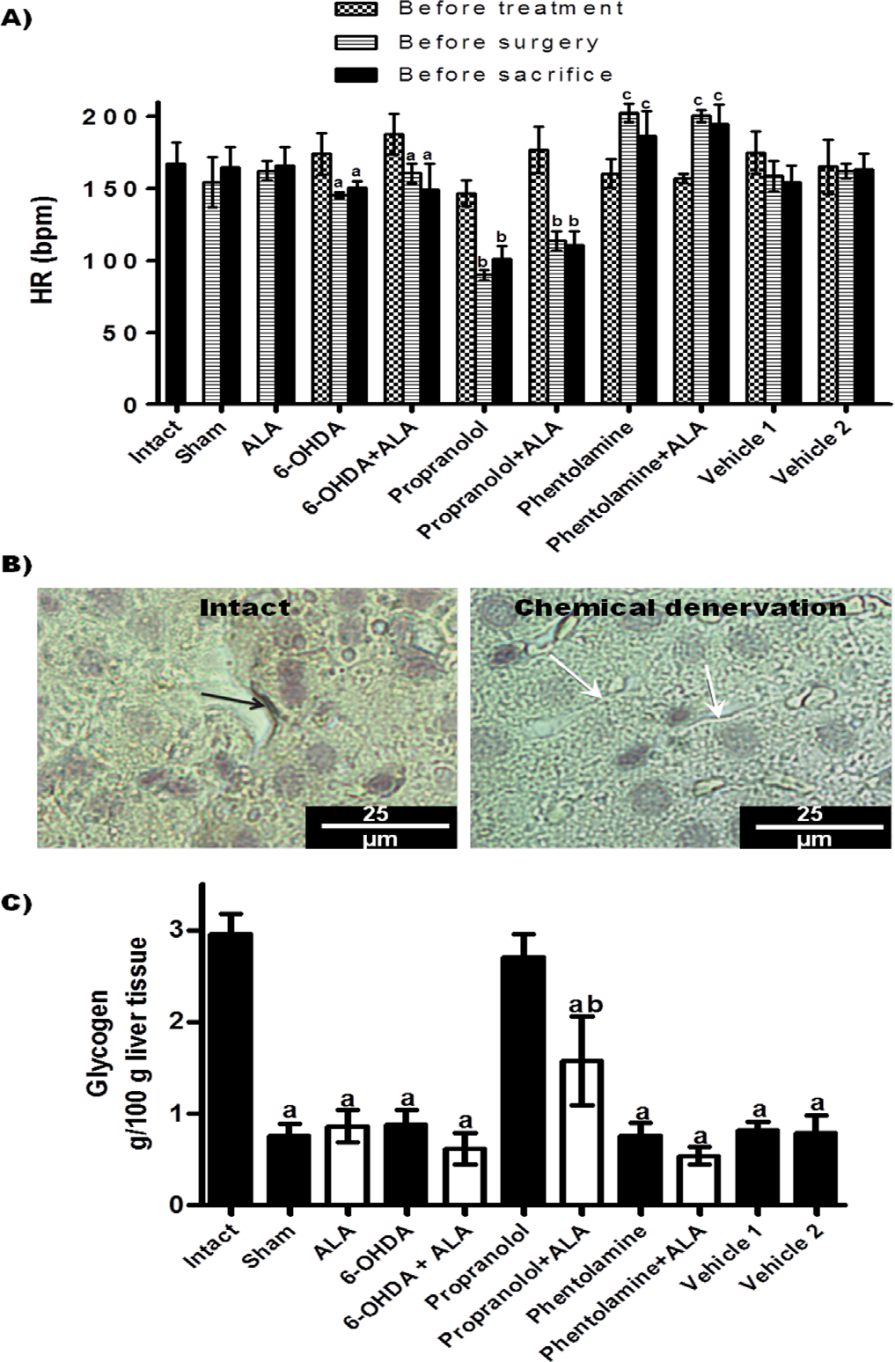
Evaluation of the autonomic nervous system. (A) Heart rate (HR) of experimental groups; each bar represents the mean value of beats per minute (bpm) ± SE (n<5); corresponding to each group, the mean values were significantly different with respect to HR measurement before starting the 6-OHDA (a), propranolol (b) and phentolamine (c) treatments, *p*<0.05. (B) Immunohistochemistry analysis of hepatic tyrosine hydroxylase (TH) synthesis: representative images of livers from animals with chemical denervation (6-OHDA and 6-OHDA+ALA groups) and healthy animals (sham and intact groups); TH(+) fiber (black arrow) and TH(-) fiber (white arrow). (C) Glycogen content in liver was determined from healthy groups (black bars) and infected with trophozoites (white bars); each bar represents the mean value of experiments performed in duplicate assays ± SE (n<5). a, Mean values significantly different from the intact group, *p*<0.05; b, mean values significantly different from the propranolol group, *p*<0.05.

In the present study, we evaluated the HR of all animals (Figure 1A). The 6-OHDA, 6-OHDA + ALA, propranolol and propranolol + ALA groups showed reduced beats per minute (bpm) before surgical and sacrifice procedures, while the phentolamine and phentolamine + ALA groups showed significant increases in the bpm prior to infection, and values remained increased until sacrifice. Immunohistochemistry showed that sympathectomized animals with 6-OHDA had inhibited TH presence in the liver, while TH (+) fibers were observed in the healthy controls (representative pictures, Figure 1B). In addition, liver glycogen content was markedly reduced in the animals under surgery, except in the animals pre-treated with propranolol (Figure 1C).

### 3.2 Modulation of amebic liver infection by the sympathetic nervous system

**Figure 2:**
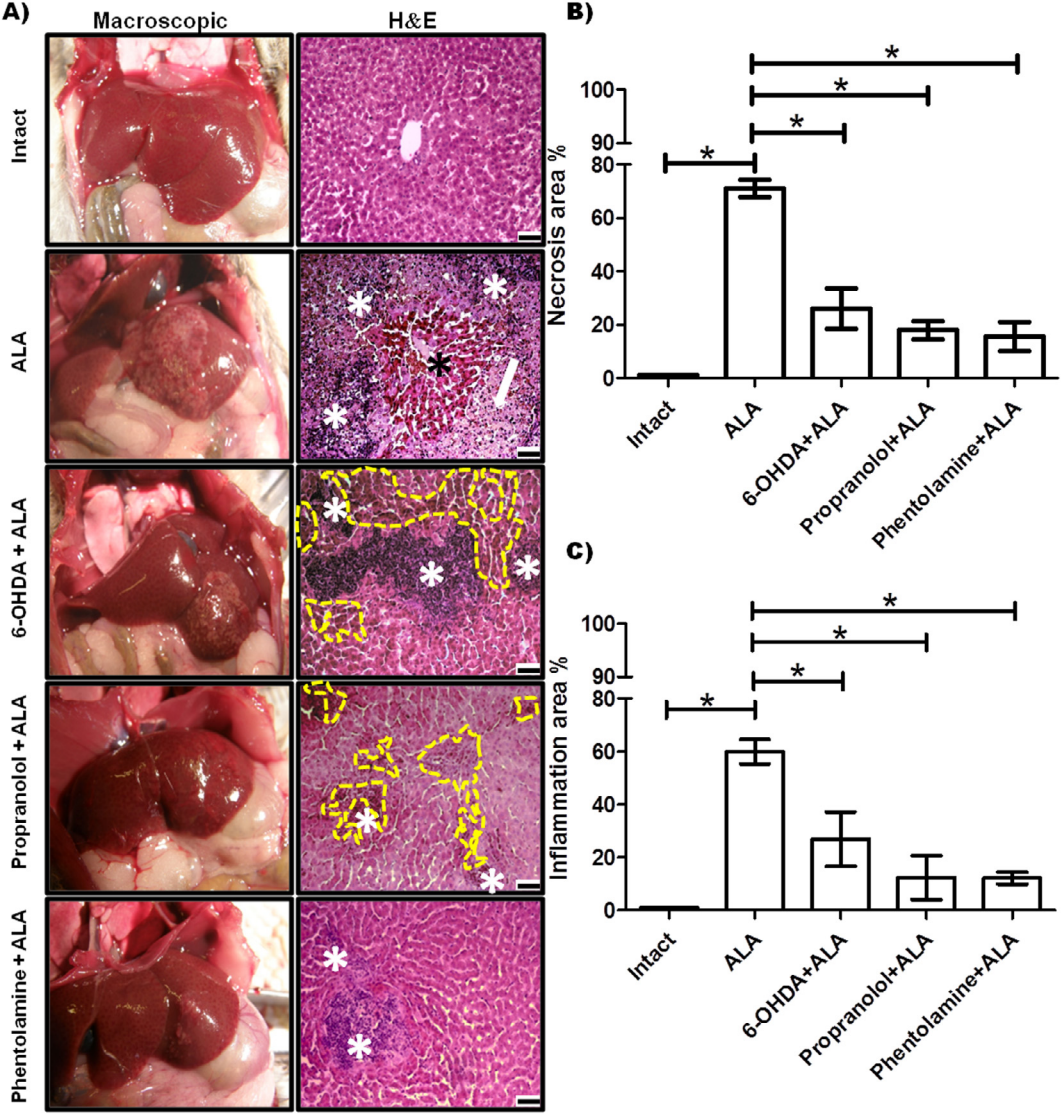
Evaluation of α and β-AR blockers during acute amebic liver infection in the hamster. (A) Representative pictures of healthy controls (intact), infected animals (ALA group), hamsters infected and pre-treated with 6-OHDA, propranolol and phentolamine (6-OHDA+ALA, propranolol + ALA, phentolamine + ALA, respectively). The hematoxylin and eosin stain (H&E) shows inflammatory infiltrates (white asterisk) and necrotic areas (white arrow) with islands of normal hepatic parenchyma (black asterisk). The 6-OHDA + ALA and propranolol + ALA groups showed hemorrhagic (dotted yellow line) and inflammatory cells (white asterisk); and phentolamine + ALA showed small areas of inflammatory cells (white asterisk). Scale bars, 50 μm. (B) Quantification of necrotic areas. (C) Quantification of inflammatory areas. * *p*<0.05 (five animals per group).

**Figure 3:**
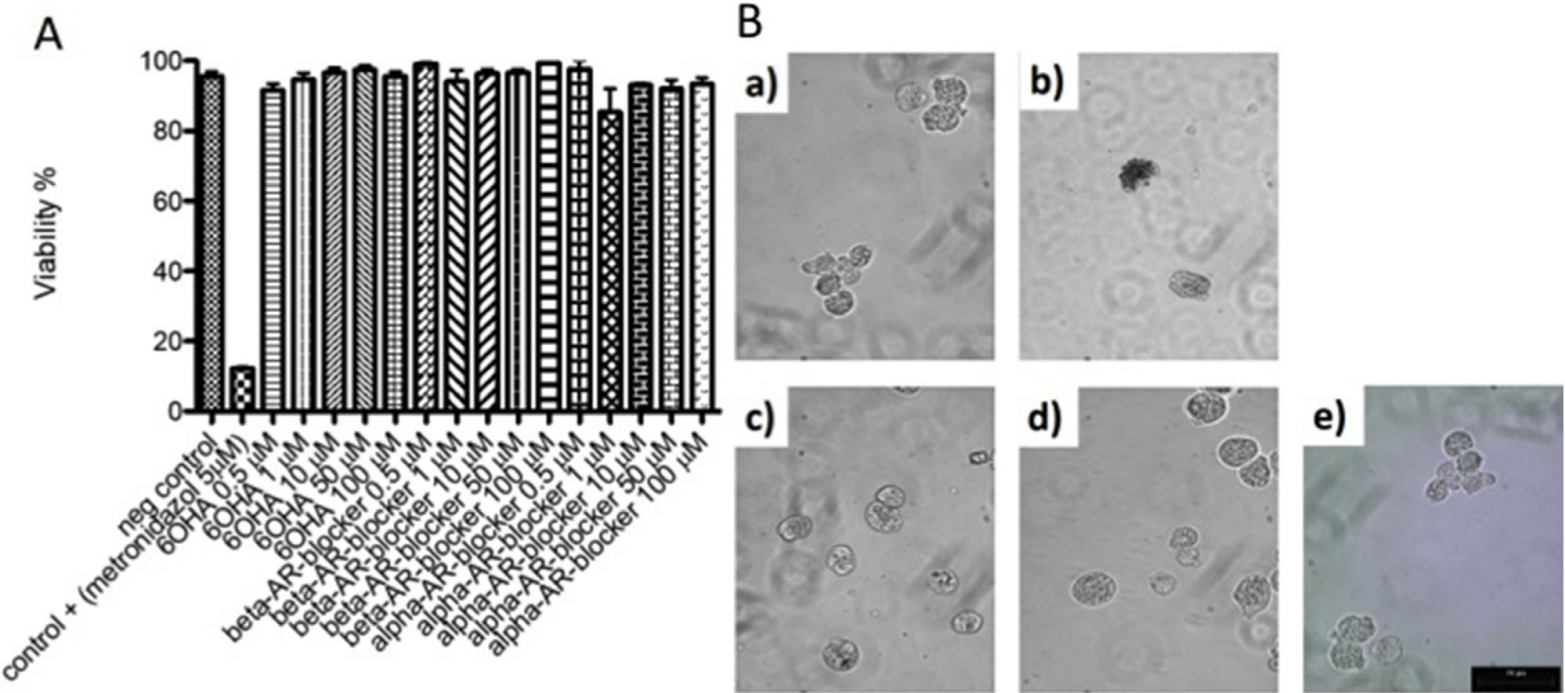
Evaluation of cell viability. A) *E. histolytica* strain (1 × 10^5^ per treatment) were incubated in Diamond medium at 37°C for 12 h with different concentrations of 6-OHDA, β-AR blocker or α-AR blocker (0.5, 1, 10, 50 and 100 μM). The cells were quantified by the Trypan Blue method. B) The figures of trophozoites of *E. histolytica* are shown with the different treatments a) control negative (free of chemicals), b) control positive (metronidazole, 5 µM), c) 6-OHDA, d) β-AR blocker, e) α-AR blocker.

Representative pictures of morphological changes in hamster livers are shown in Figure 2A. Healthy controls were morphologically normal. The ALA group showed amebic abscess visible at 12 h in all infected animals, and histological observations with H&E staining showed lesions presenting as delimited nodular necrosis areas consisting of inflammatory infiltrate surrounded by normal tissue. 6-OHDA administration before amebic infection reduced the size of the ALA and showed various small amebic lesions and hemorrhagic foci as compared to the ALA group. The livers of animals treated with propranolol appeared to show sparse damage and smaller granulomas consisting of inflammatory infiltrate and smaller hemorrhagic areas as compared to the 6-OHDA + ALA group. The phentolamine + ALA group showed few and small amebic lesions on the liver surface, and H&E staining showed scarce amebic lesions consisting of inflammatory infiltrate. In addition, in the ALA group, we observed an increased occurrence of necrotic and inflammatory areas (71 ± 3.18% and 59.85 ± 4.6%, respectively) as compared to the intact group, while the 6-OHDA + ALA group showed a lower percentage in the incidence of both areas (36 ± 7.6% and 26.88 ± 10.3%, respectively). Few necrotic and inflammatory zones were found in the propranolol + ALA group (17.94% ± 3.5 and 12.39 ± 8.3%, respectively) and the phentolamine + ALA group (15.63% ± 5.5 and 12.18 ± 2.25%, respectively) (Figures 2B, 2C). In addition, 6-OHDA and α and β-ARs blockers had no effect on the viability of *E. histolytica* at 0.5, 1, 10, 50 and 100 μM (Figure 3).

### 3.3 Enzymatic activities of ALT and γ-GTP

**Figure 4:**
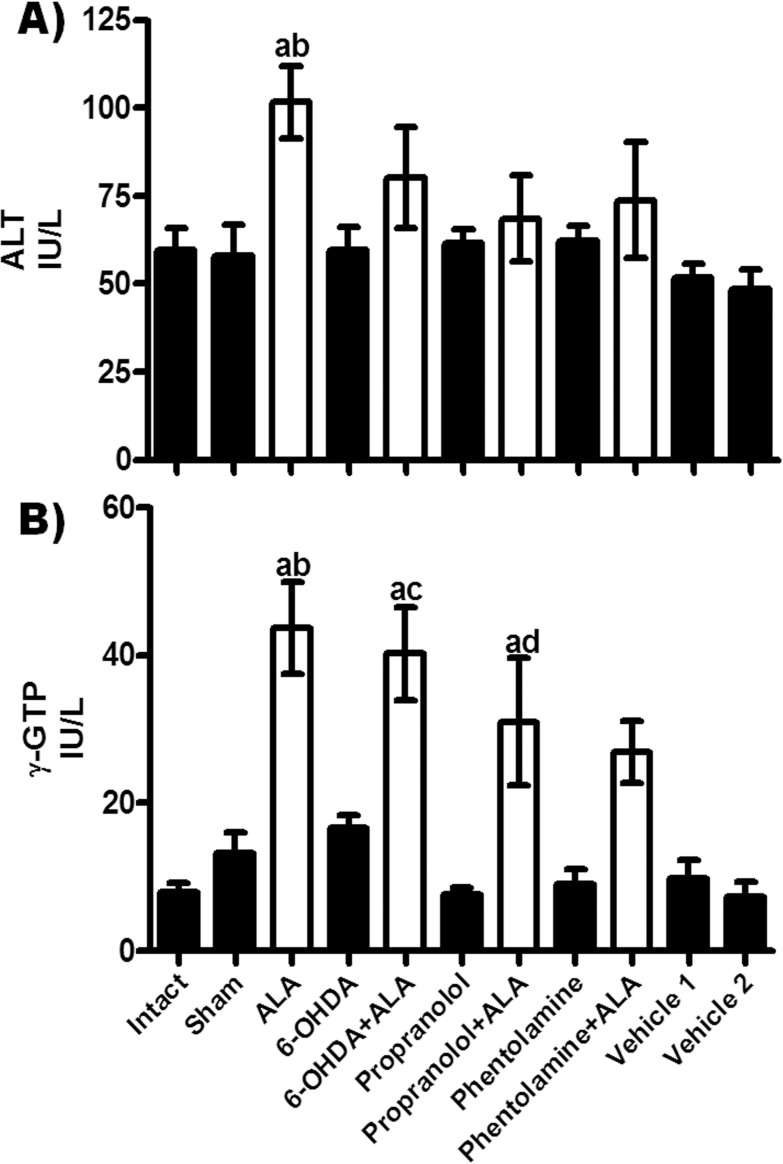
Enzymatic activities of alanine aminotransferase (ALT) and γ-glutamyl transpeptidase (γ-GTP) were determined from healthy controls (black bars) and infected animals with trophozoites (white bars). Each bar represents the mean value of experiments performed in duplicate assays ± SE (n<5). a, Mean values significantly different from the intact group, *p*<0.05; b, mean values significantly different from the sham group, *p*<0.05; c, mean values significantly different from the 6-OHDA group, *p*<0.05; d, mean values significantly different from the propranolol group, *p*<0.05.

ALT activity was elevated in serum at 12 h after amebic infection (ALA group) as compared to healthy groups, and chemical treatments showed a tendency to decrease this level (Figure 4A). Moreover, γ-GTP activity was significantly enhanced as compared with healthy controls; the 6-OHDA + ALA and propranolol + ALA groups showed tendencies to decrease γ-GTP compared to the ALA group, while the phentolamine + ALA group showed no significant difference with the healthy controls and ALA group (Figure 4B).

### 3.4 Oxidative stress during amebic liver infection

**Figure 5:**
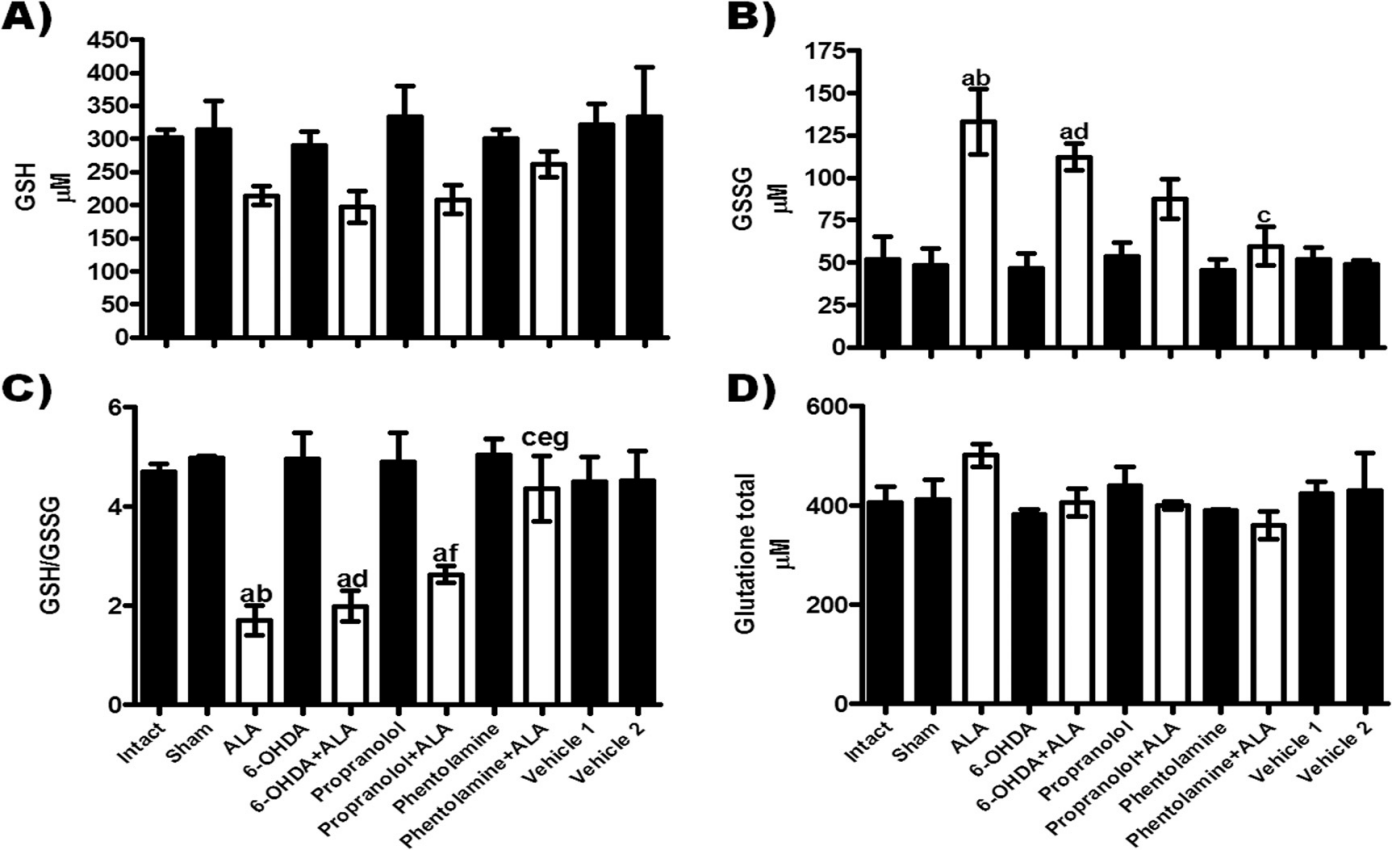
Relationship between the adrenergic system and glutathione status during ALA. Glutathione: (A) reduced GSH; (B) oxidized GSSG, (C) GSH/GSSG ratio and (D) total glutathione (GSH+GSSG), were determined in liver samples from healthy groups (black bars) and infected with trophozoites (white bars). Results are shown as the mean value of 5 animals ± SE. a, mean values significantly different *vs.* the intact group, *p*<0.05; b, mean values significantly different *vs.* the sham group, *p*<0.05; c, mean values significantly different *vs.* the ALA group; d, mean values significantly different *vs.* the 6-OHDA group, *p*<0.05; e, mean values significantly different *vs.* the 6-OHDA+ALA group, *p*<0.05; f, mean values significantly different *vs.* the propranolol group, *p*<0.05 and g, mean values significantly different *vs.* the propranolol + ALA group, *p*<0.05.

Hepatic oxidative stress was evaluated by measuring GSH, GSSG, GSH/GSSG and GSH+GSSG during ALA in hamsters (Figure 5). GSH levels were not significantly different between groups, although the groups infected with *E. histolytica* registered a tendency to decreased levels in relation to healthy controls (Figure 5A). GSSG was significantly increased in the ALA group in contrast with healthy controls; the 6-OHDA + ALA group showed a tendency to decreases in this parameter with respect to the ALA group, while the propranolol + ALA group also showed a tendency to a reduction in this parameter, but it was not statistically different from the healthy controls. Phentolamine + ALA treatment prevented this increase (Figure 5B). The GSH/GSSG ratio was significantly reduced in the ALA, 6-OHDA + ALA and propranolol + ALA groups with respect to healthy controls, while in the phentolamine + ALA group, treatment with α adrenergic stimulant prevented the decrease in the GSH/GSSG ratio induced by the amebic infection (Figure 5C). Total glutathione showed no significant alteration in any group (Figure 5D).

### 3.5 Sympathetic activity on the Nrf2 and NF-κB during amebic infection

**Figure 6:**
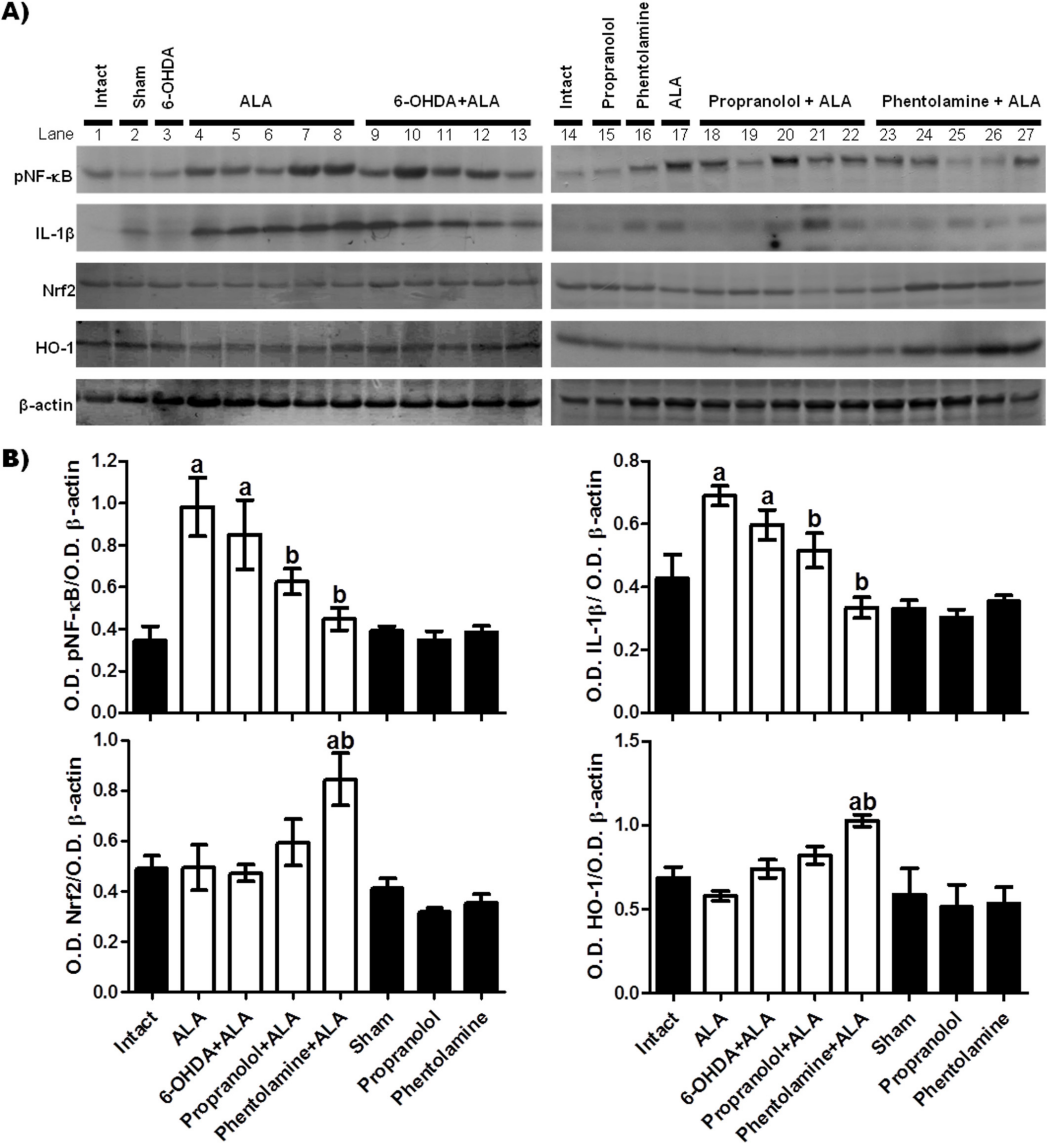
Effect of α and β-AR blockers on the protein expression of pNF-κB, IL-1β, Nrf2 and HO-1 in liver samples taken from amebic abscess areas. (A) Representative images of western blot; each line corresponds to a sample from a different animal, line 1: samples from one animal (intact group), line 2: one animal (sham group), line 3: one sample (6-OHDA group), lines 4 to 8: samples of five animals (ALA group), lines 9 to 13: samples of five hamsters (6-OHDA+ALA group), line 14: one sample (intact group), line 15: one sample (propranolol group), line 16: one sample (phentolamine group), line 17: one sample (ALA group), lines 18 to 22: samples of five animals (propranolol+ALA), lines 23 to 27: samples of five hamsters (phentolamine+ALA). (B) Signal intensities were determined by densitometric analysis of treated blots and values calculated as the ratio of each protein/β-actin. The results shown represent the mean value obtained ± SE of three independent experiments of proteins samples from the 5 hamsters. Corresponding to each protein: a, mean values significantly different from the intact group; and b, mean values significantly different from the ALA group; (*p*<0.05).

In this study, we evaluated the role of Nrf2 and NF-κB in ALA, focusing on their modulation by the sympathetic nervous system. Figure 6 shows western blots of healthy tissue from the healthy control groups (intact, sham, 6-OHDA, propranolol, phentolamine) and of liver samples that were taken from amebic abscess areas obtained from the five animals infected in each group (ALA, 6-OHDA + ALA, propranolol + ALA, phentolamine + ALA). The acute liver amebic infection with *E. histolytica* (ALA group) induced NF-κB activation and this led to increased expression of IL-1β and showed no significant changes in the expression of Nrf2, while HO-1 was reduced as compared to healthy controls, but values were not statistically different. However, the 6-OHDA + ALA group showed increased pNF-κB and IL-1β production as compared to healthy groups, but showed a tendency to decreased values with respect to ALA animals. The western blots of the propranolol + ALA group showed inhibition of NF-κB activation, whereas IL-1β expression dropped without being statistically significant with respect to the intact group. Likewise, Nrf2 and HO-1 expressions were not statistically different to healthy controls. Similarly, the phentolamine + ALA group showed decreased pNF-κB and IL-1β expressions in relation to the ALA group, and showed significant overexpression of Nrf2 and HO-1 as compared to healthy controls and the ALA group. These protein levels remained virtually unchanged in all healthy control animals.

## 4 Discussion

Our results show that amebic liver lesions are visible at 12 h, which was consistent with increased serum enzyme activity for ALT and γ-GTP; these results agreed with previous findings [1]. ALT is an enzyme located in the cytosol of hepatocytes [49], and in the present study, we observed slightly elevated levels, possibility because serum evaluation was performed at 12 h post-liver infection (ALA group). Moreover, γ-GTP activity was elevated approximately 3-fold with respect to healthy controls; this enzyme is localized in the canaliculi of hepatocytes and in biliary epithelial cells, and its increase is related to hepatic bile duct injury [20]. In addition, serum γ-GTP is also considered a marker of oxidative stress because it is responsible for the extracellular catabolism of the antioxidant GSH, which facilitates the generation of ROS [66]. ROS have been implicated in the etiology of amebiasis because they are generated by inflammatory cells [27]; however, it is not known whether γ-GTP is a contributor to development of amebiasis. Therefore, we hypothesize that increased γ-GTP activity is higher than ALT activity because: a) the increase of γ-GTP activity can be due to liver damage associated with bile duct injury, probably due to the parasite load that was administered at a specific site through intrahepatic puncture, unlike an intraportal infection; b) γ-GTP is increased to favor oxidative stress by generating ROS; and c) γ-GTP is induced as part of the antioxidant response to oxidative stress induced by the amebic infection. In addition, patients with autoimmune cholestatic diseases and autoimmune hepatitis show mildly raised aminotransferases levels, whereas γ-GTP levels are often markedly raised [19]. Importantly, macroscopic observations and H&E stains showed that chemical sympathectomy and α and β adrenergic blocking agents prevented liver damage induced by *E. histolytica* to different degrees; propranolol and phentolamine significantly prevented ALA progression in relation to 6-OHDA, but phentolamine showed superior protection as compared to propranolol. Propranolol and phentolamine provided a high degree of protection against necrosis and inflammation induced by *E. histolytica*. This suggests that the adrenergic system regulates the acute phase of amebic liver infection in the hamster.

Additionally, our study aimed to evaluate the effect of 6-OHDA, propranolol and phentolamine in relation to the oxidative stress generated during the early stage of amebic liver infection. It is known that at 12 h of amebic liver infection, the amount of polymorphonuclear leukocytes infiltrated and the lesion size are increased, forming microabscesses [63]. In addition, in a previous study [1], we reported that at this time, the increase in oxidative stress is greatest and the NF-κB pro-inflammatory pathway is activated. Moreover, the effect of the SNS and PNS during the early and late stages of amebic infection has also begun to be studied [3,42,51], but there is little knowledge on their participation and their relationship with the oxidative stress that is generated during the first hours of amebic infection. In 2011, Muñoz-Ortega [41] reported that liver parasympathectomy resulted in a larger ALA size, and a greater production of collagen fibers, causing an increase in the response against infection. This suggests that parasympathetic denervation could favor the innervations of the liver by the SNS, which then takes control of the immune response by stimulating the conversion of macrophages to epithelioid cells. In 2015, Sánchez-Alemán [51] claimed that vagotomy in hamsters increased the activity of transcriptional factor NF-κB in neutrophils and macrophages during ALA. In the same year, Ávila-Blanco [3] reported that chemical sympathectomy decreased macrophages and neutrophils positive to pNF-κB, inducing an anti-inflammatory state during the amebic infection.

In the present work, 6-OHDA treatments decreased HR, which was consistent with the findings in other studies [28] and showed the absence of TH (+) fibers in the liver, confirming sympathetic denervation. The liver tissue samples from the infection site in 6-OHDA + ALA demonstrated small lesions surrounded by hemorrhagic areas, and protein expression of pNF-κB and IL-1β were increased as compared to healthy controls, but 6-OHDA + ALA also manifested a tendency to decrease them in comparison to the ALA group, and these results were consistent with previous findings [3]. Additionally, our present results showed that chemical denervation did not increased protein expression of Nrf2 and HO-1, as compared to healthy controls. Recently, our group reported that during acute liver infection by *E. histolytica,* the Nrf2 pathway was inactivated, which may favor overactivation of NF-κB and the progression of liver damage [1]. Therefore, our results suggest that chemical sympathectomy reduced ALA progression as a result of a compensatory response by activating intrinsic autoregulatory mechanisms controlled by the PNS, although more studies are needed to confirm this hypothesis. Previously, other studies have reported that chemical denervation increased numbers of oval cells, which are resident hepatic stem cells that promote liver regeneration and repair [44]

Moreover, we evaluated the participation of adrenoreceptors (ARs) which are G-protein-coupled receptors of three major types: α1, α2, and β [6]. β-ARs can be divided into three subtypes: β1, β2 and β3. Studies have documented the presence of β1- and β2-ARs in rodent and human liver tissues [18]. In the present work, we used propranolol, a synthetic nonselective β1 and β2-AR blocker [35], which reduced HR, and prevented hepatic glycogen depletion in the propranolol group, whereas the propranolol + ALA treatment partially prevented glycogen depletion. This effect could be due to β-ARr blockade on the one hand, and cell damage generated by the parasite on the other. It has been indicated that hepatic glycogenolysis may be mediated by either α- or β-ARs, depending on the species or the state of nutrition, and not only β-ARs as previously thought [52]. However, in the present study phentolamine did not prevent glycogen depletion in the hamster liver. Several studies describe a reduction of α-adrenergic glycogenolysis and an enhancement of β-adrenergic-mediated glycogenolysis in hepatocytes from hypothyroid [38] and adrenalectomized rats [11]. Additionally, reports available in the literature on propranolol-precipitated hypoglycemia in patients suggest that the β-AR blocking agent produces hypoglycemia primarily through the depression of liver glycogenolysis [48]. Likewise, 6-OHDA did not prevent glycogen depletion. This result was in line with previous reports indicating that modifications elicited by exercise in 6-OHDA-treated rats decrease glycogen content in the liver [13]. Additionally, the groups undergoing surgical procedures (sham, ALA, vehicle 1, vehicle 2 groups) showed significant reductions in hepatic glycogen. These results are consistent with previous reports from our group showing that healthy hamsters that underwent surgery presented the "Ebb phase", demonstrated by depletion of hepatic glycogen content at 12, 24 and 36 h post-surgery, which was restored at 48 h [1].

On the other hand, three subtypes of α1-adrenoreceptors (α1-ARs) are known: α1a/A, α1b/B and α1d/D [6,25]. However, in the liver, one α1-AR subtype dominated in each species, *e.g.* humans, cats, dogs and rabbits expressed the α1A-AR, but the rat, mouse and hamster expressed the α1B-AR [17]. In addition to its role in metabolism, hepatic α1-AR is also involved in regulation of hepatocyte proliferation and liver regeneration after hepatic injury or partial hepatectomy and plays an important role in the sympatho-adrenal response to stress, such as peripheral vasoconstriction and increased cardiac contractility [10]. *In vitro* experiments with selective α-AR inhibitors, show that α2A-ARs on Kupffer cells are responsible for increased cytokine release [40]. Phentolamine is an α1AR and α2AR selective adrenergic receptor antagonist [21], which in the present study significantly prevented liver damage induced by *E. histolytica*. Therefore, we speculate the α-AR contributes to progression of amebic infection in hamsters. Various studies have suggested that α-AR activation augments pro-inflammatory cytokine production [33,56,57] and the activation of β_2_-AR results in immunosuppression by decreasing the release of pro-inflammatory cytokines [54,57]. Our results showed that phentolamine treatment significantly reduces oxidative stress (Figure 6A), increases Nrf2 and HO-1 expression (anti-oxidant system), and reduces pNF-κB and IL-1β production (pro-inflammatory system), suggesting that activation of α-ARs is necessary for progression of liver damage induced by *E. histolytica*. The inflammatory process must be present for *E. histolytica* to damage the liver [45]. At 12 h post-infection, this inflammatory response was constituted mainly of neutrophils [63], which are the major producers of oxygen-free radicals (respiratory burst). In addition, the oxidative stress induces the activation of the NF-κB pathway, resulting in the production of pro-inflammatory cytokines and in turn the Nrf2 pathway is inhibited, which exacerbates liver damage [1]. In our work, we postulate that the inhibition of NF-κB activation is induced by the prevalence of β-ARs due to the blockade of the α-ARs, resulting in natural regulation of oxidative stress, due to the resolution of inflammation and cell damage. It has been postulated that the prolonged stimulation of the SNS leads to increased inflammation with a corresponding decrease in the ability to fight infections [29]. The α-ARs in immune cells possess a pro-inflammatory effect on immune system responses, which is consistent with many chronic inflammatory disease states [47]. Based on the above, it has been shown that by blocking α1-ARs that are expressed on the surface of immune cells, the intensity of the immune response decreases, while the oxidative stress generated is also diminished [22]. The stimulation of β-ARs has been reported as a cause of generation of ROS in mitochondria [26]. The use of β-AR blockers has been indicated to reduce oxidative stress in cardiac failure [31,32]. Chemical sympathectomy or α-AR antagonist treatment has profound inhibitory effects on CCl4-induced hepatic oxidative injury, which has important implications for understanding how the response to liver injury may be controlled [34]. Moreover, our results showed that the group infected with *E. histolytica* and treated with phentolamine increased HO-1 expression. This result suggests that: A) blocking α-ARs results in an increase in β-AR activity, which is known to mediate the production of pro-inflammatory cytokines [57], but also, the β1-AR agonists mediate HO-1 induction *via* Nrf2 translocation in neonatal rat cardiomyocytes [64], as well as β2-ARs agonists in the rat liver [60]; B) the predominance or potentiation of the PNS induces an anti-oxidative and anti-inflammatory effect protecting from liver damage by this parasite. However, further studies are needed to understand in more detail the role of phentolamine over Nrf2/HO-1 in the pathogenesis of amebiasis.

In the present study, we observed that propranolol treatment also reduced pNF-κB and IL-1β in relation to the ALA group, which is consistent with other work that indicated that β-adrenergic receptors may mediate production of pro-inflammatory cytokines in the brain [65]. However, this β-AR blocking agent was not able to attenuate oxidative stress and increase the expression of Nrf2 and HO-1, suggesting that hepatic protection is mediated by decreasing the pro-inflammatory pathway of NF-κB. It is known that adrenaline may selectively protect mesenchymal C3H10T1/2 cells from oxidative stress through a mechanism related to the promoted biosynthesis of glutathione in association with transient Nrf2 expression after activation of β2-ARs [59].

## 5 Conclusion

Our study provides evidence of adrenergic regulation of inflammatory and antioxidant systems during the acute phase of ALA in the hamster. The results obtained in the present study suggest that phentolamine provides superior protection compared to propranolol, suggesting that α-ARs even more than β-ARs may be mainly involved in the pathophysiology of ALA, although it is necessary to continue the individual evaluation of each subtype of AR. However, we can also hypothesize that blockade of α and β-ARs as well as chemical denervation give rise to the predominance or potentiation of the PNS, inducing an anti-oxidative and anti-inflammatory effect protecting from liver damage by *E. histolytica*. These data reinforce the importance of studying the SNS and PNS in parasitic liver diseases.

## Conflicts of interest

The authors declare that they have no conflicts of interest in relation to this article.
